# Prediction of musculoskeletal pain after the first intravenous zoledronic acid injection in patients with primary osteoporosis: development and evaluation of a new nomogram

**DOI:** 10.1186/s12891-023-06965-y

**Published:** 2023-10-25

**Authors:** Xiaoxia Zheng, Jiangnan Ye, Qunzhang Zhan, Weifeng Zhao, Zhongqin Liao, Xiaolin Ye, Chongzheng Qu

**Affiliations:** 1 Guangzhou University of Traditional Chinese Medicine, Guangzhou, Guangdong China; 2 Chengdu University of Traditional Chinese Medicine, Chengdu, Sichuan China; 3 The Third Affiliated Hospital of Guangzhou University of Traditional Chinese Medicine, No.261, Longxi Avenue, Guangzhou, 510000 Guangdong China

**Keywords:** Zoledronic acid, Osteoporosis, Pain, Nomogram, Acute phase reaction

## Abstract

**Objective:**

To construct a new prediction nomogram to predict the risk of musculoskeletal pain in patients with primary osteoporosis who receive zoledronic acid intravenously for the first time.

**Method:**

Clinical data of 368 patients with primary osteoporosis who received the first intravenous injection of zoledronic acid in our hospital from December 2019 to December 2022 were studied. Patients were divided into a musculoskeletal pain group (n = 258) and a non-musculoskeletal pain group (n = 110) based on the presence or absence of musculoskeletal pain 3 days after injection. Statistically significant predictors were screened by logistic regression analysis and the minimum absolute contraction and selection operator (LASSO) to construct a nomogram. The nomogram was evaluated by the receiver operating characteristic (ROC) curve, the calibration curve, the C-index, and the decision curve analysis (DCA) and verified in a validation cohort.

**Results:**

The independent predictors of the nomogram were age, serum 25-hydroxyvitamin D, NSAIDs, prior Vitamin D intake, and BMI. The area under the ROC curve (AUC) was 0.980 (95% CI, 0.915–0.987), showing excellent predictive performance. The nomogram c index was 0.980, and the nomogram c index for internal verification remained high at 0.979. Moreover, calibration curves show that the nomogram has good consistency. Finally, the DCA showed that the net benefit of the nomogram was 0.20–0.49.

**Conclusion:**

Musculoskeletal pain is a common symptom of APR in OP patients treated with intravenous zoledronic acid. Risk factors for musculoskeletal pain after zoledronic acid injection in OP patients were: non-use of NSAIDs, youth (<80 years old), serum 25 (OH) D<30ng /mL, no prior intake of vitamin D, BMI<24 kg /m^2^. A nomogram constructed from the above predictors can be used to predict musculoskeletal pain after the first zoledronic acid injection.

**Supplementary Information:**

The online version contains supplementary material available at 10.1186/s12891-023-06965-y.

## Introduction

The incidence of primary osteoporosis is high in the elderly population around the world. The prevalence of osteoporosis in elderly women over 70 years old in China is 51.3%, and that in elderly men is 12.3% [1]. The prevalence of osteoporosis has shown an upward trend in the past 10 years, accompanied by a higher probability of vertebral fractures and clinical fractures [1]. A study on the medical costs of osteoporosis-related hip fractures (HF) suggests that the burden of managing HF in Asia is heavy, accounting for 18.95% of the per capita GDP of each country in 2014 [2]. Treatment and management measures for osteoporosis have become more important [3]. Bisphosphonates are first-line anti-osteoporosis drugs [4, 5]. Studies have shown that adherence to 3–5 years of oral alendronate or intravenous zoledronic acid is an effective measure to reduce the risk of fracture in patients with osteoporosis [6]. Compared with other bisphosphonates, patients showed preference for the treatment of intravenous zoledronate every year due to the convenience of medication and weak gastrointestinal stimulation [7]. Unfortunately, although zoledronic acid injection therapy is only given once a year, there are still two-thirds of patients who refuse the second year of injection therapy, and the overall compliance of osteoporosis patients with bisphosphonate therapy is still low [8, 9]. The low compliance of patients with bisphosphonates inevitably leads to low efficacy of anti-osteoporosis treatment. The efficacy of bisphosphonates in reducing the risk of fracture has not been fully exerted, and medical resources are seriously wasted [2, 10]. In view of the above problems, current studies have found that acute phase adverse reactions (APR) and insufficient explanation of intravenous medication are the main causes of low compliance with intravenous zoledronic acid [8]. Analysis of APR-related risk factors and preventive measures is the key to improving the efficacy of bisphosphonates in the treatment of osteoporosis.

As the most common adverse event after intravenous injection of zoledronic acid, APR is composed of different symptoms such as musculoskeletal pain, fever, and eye inflammation [11]. Studies have suggested that the actual probability of APR in clinical practice is higher than that reported in clinical trials [12]. Among them, the probability of occurrence or aggravation of musculoskeletal pain was as high as 36.46%, and the incidence of fever was 28.65% [13]. Musculoskeletal pain, as a high-risk symptom of APR after zoledronic acid injection, is listed as a potential side effect by the medication instructions of all bisphosphonates, which increases the pain and fear of elderly patients after medication. It seriously affects patients’ willingness to use drugs and is an important factor leading to low compliance with bisphosphonates. Therefore, it is necessary to predict and prevent musculoskeletal pain symptoms after a zoledronic acid injection.

At present, there are few predictive tools for predicting APR risk after intravenous injection of zoledronic acid. Few studies have focused on predicting the fever symptoms of APR [14]. APR is a symptom cluster represented by fever, musculoskeletal pain, and other symptoms, and it is insufficient to predict only fever symptoms. It is necessary to predict the symptoms of musculoskeletal pain in APR, considering the high incidence of musculoskeletal pain. As far as we know, there is no predictive model or analysis tool for the musculoskeletal pain symptoms of APR. This study aims to establish a predictive model for the risk of musculoskeletal pain in patients with primary osteoporosis after intravenous injection of zoledronic acid. Make up for the shortcomings of existing APR prediction tools. A reliable and comprehensive predictive model will help clinicians assess musculoskeletal pain symptoms, promote the proper use of zoledronic acid, and ultimately improve the compliance of OP patients with zoledronic acid treatment. Therefore, we are committed to developing a reliable and accurate risk prediction model.

## Method

### Patients

This study was based on the clinical data of 368 patients diagnosed with primary osteoporosis in the Third Affiliated Hospital of Guangzhou University of Traditional Chinese Medicine from December 2019 to December 2022. These patients were diagnosed with OP and treated with intravenous zoledronic acid. Diagnostic criteria for primary osteoporosis are: bone mineral density less than − 2.5 after excluding other metabolic bone diseases; or bone mineral density is normal or reduced but there is a brittle fracture [15, 16]. Exclusion criteria are as follows: 1.Non-first intravenous injection of zoledronic acid or previous history of bisphosphonates use. 2. Are allergic to zoledronic acid or bisphosphonates. 3.Local or systemic infection, other diseases can be manifested as fever. 4.Malignant tumors, muscle weakness, and other diseases that affect the judgment of musculoskeletal pain symptoms.5. Use statins and other drugs that may cause muscle pain. 6.Dementia, aphasia and other diseases that cannot cooperate with the study. 7.Clinical data is incomplete. 8. Participants who refused the injection for reasons such as medical insurance or fear of adverse reactions were also excluded. This study was approved by the Ethics Committee of the Third Affiliated Hospital of Guangzhou University of Traditional Chinese Medicine (YJ-LW-20230112-001), and subject consent was obtained according to the Declaration of Helsinki.

### Data acquisition

Patient data were collected and recorded prior to intravenous therapy: two experienced orthopedic surgeons assessed and recorded symptoms that could cause musculoskeletal pain, such as osteoarthritis, accidental trauma, surgical history, etc. Also, we will measure and record a range of relevant indicators for participants, such as calcium levels, rheumatoid factors, C-reactive protein, etc.The predictors included in the study were: gender, age, BMI, fracture, NSAIDs, serum 25 ( OH ) D, PTH, T index, fluid volume, prior Vitamin D intake, OC. The above variables were converted into dichotomous variables based on previous literature reports [13, 14, 17–22]. Specifically, age risk factors were divided into four groups: less than 60 years old, 60 to 70 years old, 70 to 80 years old, and 80 years or older. Bone mineral density was measured by experienced professionals using the American HOLOGIC, Discovery-Wi dual-energy X-ray absorptiometry for hip and lumbar bone density measurement. The lowest bone mineral density measured was evaluated for osteoporosis. In order to facilitate the description, this study combined the symptoms of bone pain and muscle pain, joint swelling, and joint pain after zoledronic acid injection treatment as musculoskeletal pain symptoms. We selected musculoskeletal pain symptoms as the dependent variable of this study to construct a prediction model. A Visual Analogue Scale / Score ( VAS ) was used to evaluate the symptoms of musculoskeletal pain before and after treatment. Whether musculoskeletal pain symptoms occur depends on the VAS score difference, that is, the VAS score after an intravenous infusion of zoledronic acid minus the VAS score before treatment. Two senior doctors in our hospital guided patients to a VAS score respectively, and the average value was recorded. We changed the VAS score difference for musculoskeletal soreness into a dichotomous variable: VAS score difference ≤ 0 meant no pain occurred, while a VAS score difference > 0 meant pain occurred. Considering that APR mostly occurs within 3 days after infusion, the final data included in the study are based on the highest VAS score difference measured within those three days.

### Statistical analysis

Data were analyzed using R software (version 4.1.0) and SPSS (version 24). Continuous variables are expressed as mean ± standard deviation, and independent sample t test or analysis of variance (ANOVA) is used for comparison between groups. The categorical variables were expressed as ratios, and the chi-square test was used for comparison between groups. The difference was defined as statistically significant at *P* < 0.05. The influencing factors of APR were preliminarily determined through literature research. Variables with univariate analysis *P* < 0.1 were included in multivariate logistic regression analysis. Multivariate logistic regression analysis *P* < 0.05 variables as independent predictors.

## Results

### Patient characteristics

A total of 368 OP patients receiving intravenous zoledronic acid treatment met the inclusion criteria. Among the 258 patients included in the training set, 118 patients had musculoskeletal pain symptoms, and the remaining 140 patients had no musculoskeletal pain symptoms. The average age of the patients in the musculoskeletal pain group was 70.92 ± 7.38 years old, and the average age of the non-musculoskeletal pain group was 82.01 ± 4.24 years old. The validation set included 110 patients: 46 patients in the musculoskeletal pain group and 64 patients in the non-musculoskeletal pain group. The average age of the musculoskeletal pain group in the validation set was 69.01 ± 5.45 years old, and the age of the non-musculoskeletal pain group was 81.77 ± 4.65 years old. The probabilities of musculoskeletal pain in the training set and the validation set were 45.6% and 42.1%, respectively. There was no significant difference in the values of patient characteristics and predictors between the training set and the validation set, as shown in Table 1 (Table 1. Patient Baseline Characteristics).

### Evaluation and screening of predictors

**Fig. 1:**
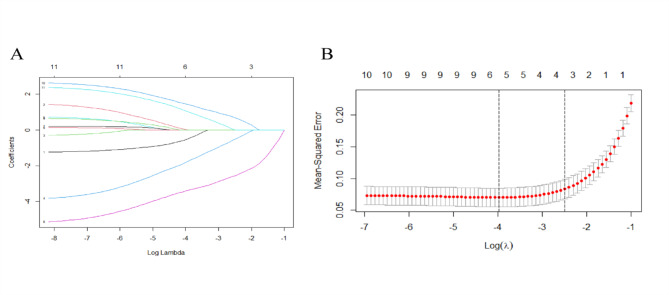
Predictor feature selection using the LASSO logistic regression model **A** Te tuning parameter(λ) was determined in the LASSO model by using a tenfold crossvalidation and a minimum criterion. In Figure A, the lower abscissa is log (lambda), the upper abscissa is the number of non-zero coefficients in the model, and the ordinate is the coefficient of the predictor. The different colored curves represent the trajectories of 11 different predictor coefficients **B** The LASSO coefcient profle plot was generated against the log (lambda) sequence. In Figure B, the bottom horizontal coordinate is the logarithm of the penalty coefficient log (λ), and the top horizontal coordinate represents the number of predictors left in the equation for different λ. The vertical coordinate is the Mean-Squared error. The dashed line on the left is λmin, representing λ with the smallest deviation. The number of predictors is 5, when the model has the highest fitting effect

After literature research, 11 possible predictors were included in univariate analysis (Table 2, univariate logistic regression table). Six variables with P values < 0.1 in univariate analysis were included in LASSO regression analysis. Finally, five variables were identified as significant predictors by ten-fold cross-validation (Fig. 1A: LASSO regression path diagram; Fig. 1B: LASSO regression minimum λ graph). Multivariate logistic regression analysis showed that age, serum 25(OH) D, NSAIDs, prior Vitamin D intake, and BMI were independent predictors of APR after zoledronic acid injection (Table 3: Multivariate logistic regression table). The selection, conversion, and classification of the above predictors are based on sufficient literature research to ensure the simplicity and effectiveness of the final model.

### Development and verification of the nomogram

**Fig. 2:**
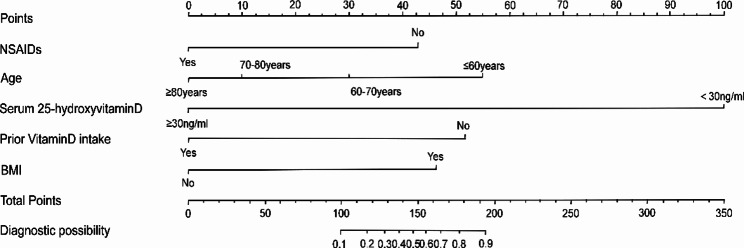
Nomogram (The risk of musculoskeletal pain in OP patients receiving zoledronic acid injection for the first time was predicted by calculating the total score of the six parameters. **NSAIDs**: “Yes” means that NSAIDs are used before injection therapy, and the score is 0. “No” means that NSAIDs have not been used before injection therapy, and the score is 42 points. **Age**: “≥80years” means that the participant is 80 years of age or older and has a score of 0. “70–80 years” means the participant is 70–80 years old and scores 10 points. “60–70 years” means participants are 60–70 years old and score 30 points. “≤60 years” means that the participant is 60 years old or less, and the score is 54 points. **Serum 25-hydroxyvitamin D**: “≥30ng/ml” represents a Serum 25-hydroxyvitamin D level higher than 30ng/ml before injection. Score 0 points; “< 30ng/ml” means that the participant had less than 30ng/ml of Serum 25-hydroxyvitamin D before the injection and scored 100 points. **Prior Vitamin D intake**: “Yes” means that participants took Vitamin D intake before injection and scored 0 points. “No” means that participants did not take vitamin D supplementation before injection therapy and scored 52 points. **BMI**: “Yes” means the participant has a BMI of less than 24 kg/m2 and a score of 45; “No” means that the participant has a BMI greater than 24 kg/m2 and a score of 0.)

**Fig. 3:**
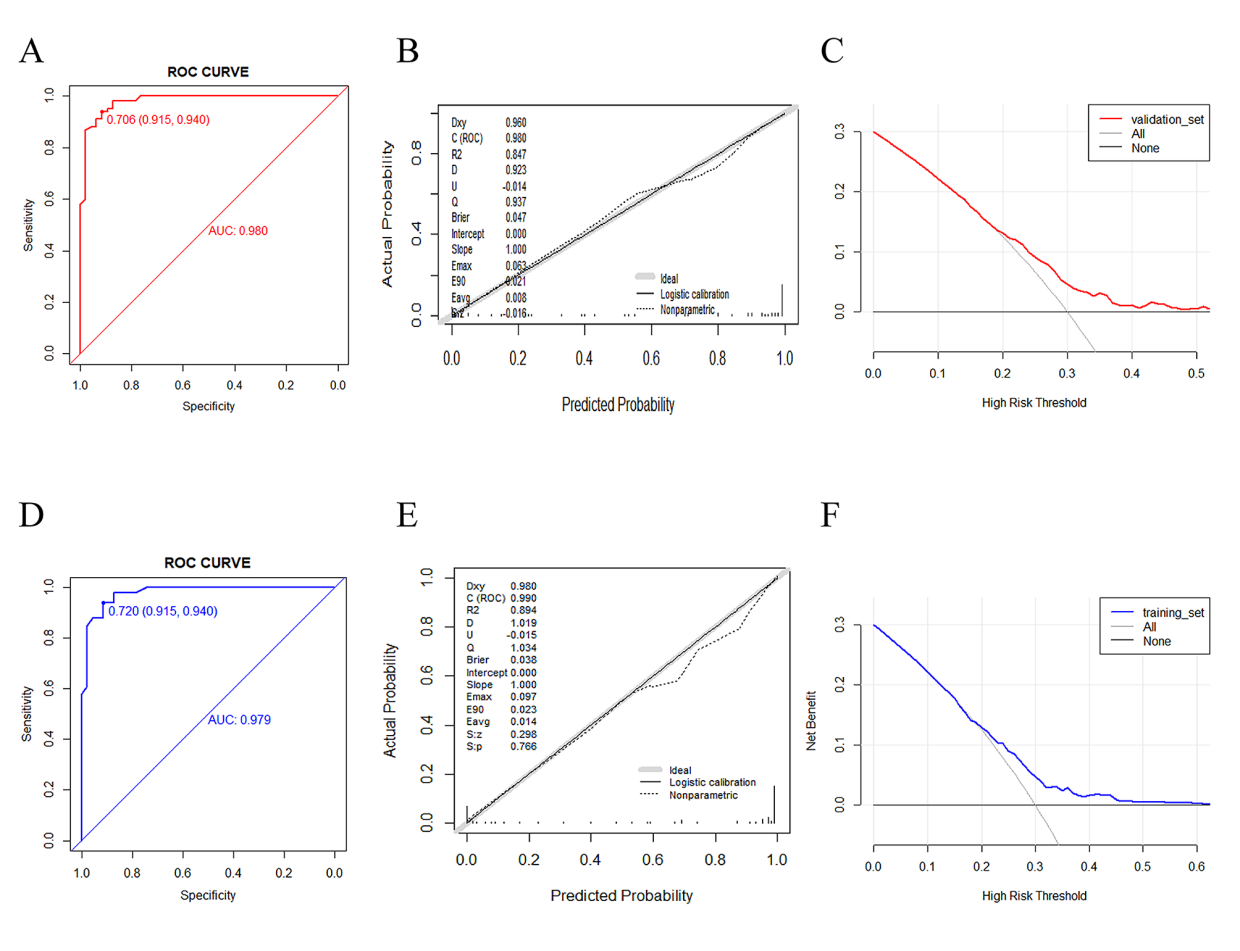
(Figure **A** ROC curve of the training set; Figure **D** ROC curve of the validation set; Figure **B** training set calibration curve; Figure **E** verification set calibration curve diagram; Figure **C** training set decision curve; Figure **F** verification set decision curve)

A nomogram of musculoskeletal pain after zoledronic acid injection was established based on five reliable predictors (Fig. 2: Nomogram). The internal verification of the model uses 1,000 bootstrapping methods. Patients included in the study were randomly divided into a training set and a validation set by 7:3 using SPSS (version 24). The training set is used to create a nomogram, and the validation set is used to validate the nomogram obtained from the training set data. This was done to evaluate the internal effectiveness of the nomogram. The receiver operating characteristic ( ROC ) curve, calibration curve, and decision curve analysis (DCA) were drawn in the training set and validation set by R software to evaluate the discrimination, calibration, and clinical utility of the model. When the nomogram constructed from the training set performs well in the verification set, it indicates that it is reliable. The discrimination of the model was evaluated by the ROC curve. In the training set, the AUC of the muscle soreness model was 0.980 (Fig. 3A: ROC curve of the training set). In the validation set, the AUC of the muscle soreness model was 0.979 (Fig. 3D: ROC curve of the validation set). The ROC curve shows that the discrimination of the model is excellent. The calibration of the model was evaluated by a calibration plot. In the training set, the calibration curve shows that the predicted results of the nomogram are highly consistent with the actual observation results (Fig. 3B: Calibration curve of the training set). In the validation set, the calibration curve fits well (Fig. 3E: Calibration curve of the validation set). The clinical applicability of the model was assessed using decision curve analysis. In the training set, the decision curve shows that the prediction model can produce a good net benefit and clinical practicability (Fig. 3C: Decision curve of the training set ). The net benefit of the muscle pain prediction model was 0.20–0.49. In the validation set, the net benefit of the muscle pain prediction model was 0.17–0.61. (Fig. 3F: Decision curve of the verification set).

### How to use Nomogram

**Usage: Risk predictor**: On the left side of this nomogram are five predictor factors, such as NSAIDs, age, prior vitamin D intake, etc. Each variable is marked with its value range on the line segment. Scores can be obtained according to the situation of the patient. **Score**: including “Points” and “Total Points”. Points refer to the score corresponding to each variable in different value ranges. Total Points is the total score obtained by adding the scores of five variables. **Diagnostic possibility**: “Diagnostic possibility “is the probability of musculoskeletal pain symptoms after injection treatment. The total score is obtained by adding the scores of the five predictors. The Diagnostic possibility can be obtained by drawing vertical lines downward according to the Total Points in the nomogram.

**Table 1:** Demographic and clinical characteristics of training sets and validation sets

predictors	Training cohort(n = 258)		*P*	Validation cohort(n = 110)		*P*
	Musculoskeletal pain(n = 118)	Non-musculoskeletal pain (n = 140 )		Musculoskeletal pain(n = 46)	Non-musculoskeletal pain (n = 64)	
**Gender,n(%)**			0.256			0.701
Male	20(17.8%)	33 (23.6%)		8 (17.4%)	13 (20.3%)	
Female	98(82.2%)	107(76.4%)		38 (82.6%)	51 (79.7%)	
**Age,years,n(%)**			0.000			0.000
< 60 60–70 70–80	5 (4.3%) 22 (18.6%) 68 (57.6%)	7 (5.0%) 16 (11.4%) 20 (14.3%)		3 (6.5%) 19 (41.3%) 13 (28.3%)	3 (4.7%) 5(7.8%) 14 (21.9%)	
>80	23 (19.5%)	97 (69.3%)		11 (23.9%)	42 (65.6%)	
**BMI, n(%)**			0.000			0.001
< 24	77 (65.3%)	57 (40.7%)		30 (65.2%)	22 (34.4%)	
≥ 24	41 (32.7%)	83 (59.3%)		16 (34.8%)	42 (65.6%)	
**NSAIDs**			0.003			0.000
Yes	15(12.7%)	115(82.1%)		7(15.2%)	51(79.7%)	
No	103(87.3%)	25(17.9%)		39(84.8%)	13(20.3%)	
**Fluidinfusion**			0.000			0.027
Yes	68 (57.6%)	92 (65.7%)		33(55.4%)	34(52.9%)	
No	50 (42.4%)	50 (42.4%)		13(44.6%)	30(47.1%)	
**Fracture**			0.668			0.977
Yes	55(46.6%)	69(49.3%)		20(43.5%)	28(43.8%)	
No	63(53.4%)	71(50.7%)		26(56.5%)	36(56.3%)	
**25(OH)D**			0.000			0.000
< 30	90(76.3%)	38(27.1%)		32(69.6%)	18(28.1%)	
≥ 30	28(23.7%)	102(72.9%)		14(30.4%)	46(71.9%)	
**PTH**			0.050			0.562
< 70	93(73.8%)	123(87.9%)		36(78.3%)	47(73.4%)	
≥ 70	25(21.2%)	17(12.1%)		10 (21.7%)	17(26.6%)	
**OC**			0.196			0.033
15–46	93(78.8%)	119(85.0%)		29(63.0%)	52(81.2%)	
≤ 15 or ≥ 46	25(21.2%)	21(15.0%)		17(37.0%)	12(18.8%)	
**T-index**			0.777			0.899
≤-2.5	72(60.0%)	83(59.3%)		25(54.3%)	34(53.1%)	
0-2.5–3.0	46(39.0%)	57(40.7%)		21(46.7%)	30(46.9%)	
**prior Vitamin D intake**			0.000			0.000
supplement	96(81.4%)	30(21.4%)		37(80.4%)	21(32.8%)	
no supplement	22(18.6%)	111(78.6%)		9(19.6%)	43(67.2%)	

**Table 2:** Univariate logistic regression table

characteristics	B	SE	OR	CI	Z	P
Gender	-0.803	0.533	0.45	0.16–1.27	-1.507	0.132
Age	-2.144	0.401	0.12	0.05–0.26	-5.353	0.000
BMI	1.667	0.381	5.29	2.51–11.17	4.369	0.000
NSAIDs	-0.718	0.361	0.49	0.24–0.99	-1.987	0.047
Fluidinfusion	0.414	0.356	1.51	0.75–3.04	1.165	0.244
Fracture	0.048	0.354	1.05	0.52–2.1	0.135	0.893
25(OH)D	-4.442	0.588	0.01	0-0.04	-7.553	0.000
PTH	0.653	0.402	1.92	0.87–4.23	1.625	0.104
OC	0.364	0.420	1.44	0.63–3.28	0.866	0.387
Tindex	0.664	0.358	1.94	0.96–3.92	1.853	0.064
prior Vitamin D intake	2.842	0.470	17.14	6.82–43.07	6.042	0.000

**Table 3:** Multivariate logistic regression table

characteristics	B	SE	OR	CI	Z	P
Age	-3.403	1.024	0.03	0-0.25	-3.324	0.001
BMI	2.134	0.846	8.45	1.61–44.36	2.523	0.012
NSAIDs	-1.979	0.933	0.14	0.02–0.86	-2.121	0.034
25(OH)D	-4.614	0.966	0.01	0-0.07	-4.777	0.000
prior Vitamin D intake	2.389	0.847	10.9	2.07–57.35	2.821	0.005

## Discussion

Musculoskeletal pain is a common symptom of APR in OP patients after intravenous injection of zoledronic acid, which is listed as a potential side effect by almost all the prescription information for bisphosphonates [23]. A multicenter international trial involving 7765 patients with osteoporosis suggested that musculoskeletal pain and fever were the most common adverse events within 3 days after infusion of 5 mg zoledronic acid or placebo. The incidence of symptoms in the two groups was about 20% in the zoledronic acid treatment group and less than 3% in the placebo group [20]. FDA recommends that clinicians pay attention to adverse reactions such as musculoskeletal pain and fever that may occur after using bisphosphonates, and consider discontinuation if necessary. Further studies are still needed to evaluate the prediction and prevention methods of adverse reactions of bisphosphonates.

We identified and screened indicators with important predictive value for APR after zoledronic acid injection, and developed a nomogram model as a tool to assist clinical diagnosis. The prediction model can assist clinicians in assessing the risk of musculoskeletal pain in OP patients receiving intravenous zoledronic acid. Therefore, it can solve the clinical difficulties of low compliance with bisphosphonates drugs, maximize the efficacy of anti-osteoporosis treatment, and ultimately reduce the fracture probability of OP patients. Whether in the training set or the validation set, the nomogram provides an excellent ROC curve for risk assessment. Physicians can identify high-risk groups of APR, explain in advance, and give reasonable suggestions. The high risk factors of musculoskeletal pain after zoledronic acid injection in OP patients are: young age (< 80 years old), BMI < 24 kg /m^2^, not using NSAIDs in advance, not supplementing vitamin D in advance, and serum 25 (OH) D < 30nmol /L. The above risk factors should be properly assessed by the clinician before providing zoledronic acid injection therapy.

At present, the potential mechanism of bisphosphonates-induced APR is still unclear. Studies have shown that bisphosphonates induces APR by inhibiting the mevalonate pathway, which can induce peripheral blood γδT cells to produce TNF α, IL6 and other pro-inflammatory cytokines [24–26]. Suppressor of cytokine signaling-3 (SOCS3) is an important regulator of various cell functions, that can regulate the production of cytokines and is closely related to the pathogenesis of diseases such as rheumatoid arthritis. Scheller found that zoledronic acid can inhibit the accumulation of SOCS3 protein, thereby enhancing the secretion of macrophage cytokines and promoting inflammatory pathological processes [25]. Considering zoledronic acid’s good bone matrix binding ability and long drug half-life, its inhibition of SOCS3 protein accumulation may explain the most common symptoms of fever and musculoskeletal pain in APR.

We found that patients older than 75 years of age have a lower probability of musculoskeletal pain, which is consistent with the results of Ding, Y [13]. We speculate that this phenomenon may be related to the decrease of cellular immune function and proinflammatory cytokine activity in elderly patients [24]. The more active the immune response, the stronger the APR induced by zoledronic acid. In addition, this study found that the probability of musculoskeletal pain in patients with a BMI higher than 24Kg / m^2^ was lower than that in patients with lower BMI and a thinner body. We speculate that it is related to the higher metabolic rate of obese patients.

In this study, patients with serum 25 (OH) D less than 30 ng/mLhad a higher risk of musculoskeletal pain, which was consistent with the results of Bertoldo Francesco et al [17, 22]. When the 25 (OH) D level is lower than 30 ng/mL, the probability of fever in patients after administration is higher than that in patients with 25 (OH) D levels higher than 40 ng / mL. Serum 25 ( OH ) D is a reliable predictor of APR. In addition, patients who received vitamin D supplementation prior to injection therapy were less likely to experience musculoskeletal pain than patients who did not receive supplementation. Considering that vitamin D is related to the processes of γt cell proliferation and cytokine production, we believe that it is beneficial to supplement vitamin D in advance for people at high risk of APR, which is consistent with the results of other scholars [17, 19]. Serum 25 (OH) D < 30 ng/mL and no vitamin D supplementation in advance are associated with a high risk of musculoskeletal pain after zoledronic acid injection. It is recommended that patients with the first zoledronic acid infusion should ensure appropriate serum 25(OH)D levels and consider the ' vitamin D supplementation period ' before infusion if necessary [19].

The results of this study suggest that prophylactic use of NSAIDs can reduce the incidence of musculoskeletal pain symptoms. Wark, J.D et al.found that oral ibuprofen could effectively control the transient influenza-like symptoms caused by 5 mg zoledronic acid, which was consistent with our results [27]. In addition, commonly used antipyretic analgesics such as acetaminophen are also effective in preventing musculoskeletal pain. The study of Reid, I.R suggested that the application of acetaminophen can significantly reduce the probability of musculoskeletal pain after zoledronic acid injection [20]. However, studies have also suggested that acetaminophen alone does not reduce the incidence of musculoskeletal pain, and dexamethasone 4 mg/d is required before and after zoledronic acid injection [27]. Although the results of multivariate analysis suggest that early use of NSAIDs can effectively reduce the incidence of musculoskeletal pain, most of the current studies on zoledronic acid-induced APR focus on body temperature indicators. There are few studies on musculoskeletal pain symptoms because they cannot be quantified like body temperature indicators. We believe that future research can focus on exploring the mechanism and prevention of musculoskeletal pain symptoms.

Interestingly, in addition to the predictors included in this study, POPP A W [28]’s study also suggested that height had a predictive effect on the occurrence of APR. Patients with a higher height (160.8 cm versus 157.9 cm) were less likely to develop APR. As we all know, OP can lead to bone structure damage, such as trabecular collapse. With the progression of the disease, OP patients may present with vertebral compression fractures, humpbacks, etc. These factors inevitably lead to a decrease in the height of patients. Therefore, we speculate that the predictive effect of height factor on APR may be caused by the correlation between the disease progression of osteoporosis and height factor.

Our research has some limitations. First, as a retrospective study, there is a certain degree of data loss. Second, the patients we studied were from the same medical institution, and no large multi-center sample study was conducted. Although the model we established has been verified by the validation set, it still cannot ensure the applicability of other races in other countries. In the future, the sensitivity and clinical applicability of the model can be further improved through multi-center retrospective validation studies or prospective randomized controlled studies.

## Conclusion

Our study found that age, BMI, serum 25 ( OH ) D, NSAIDs, and prior Vitamin D intake were independent risk factors for zoledronic acid-induced musculoskeletal pain. A nomogram containing 5 predictors of appeal can accurately predict the risk of musculoskeletal pain after zoledronic acid infusion.

### Electronic supplementary material

Below is the link to the electronic supplementary material.

Supplementary Material 1

Supplementary Material 2

Supplementary Material 3

Supplementary Material 4

Supplementary Material 5

Supplementary Material 6

Supplementary Material 7

Supplementary Material 8

Supplementary Material 9

Supplementary Material 10

Supplementary Material 11

Supplementary Material 12

Supplementary Material 13

Supplementary Material 14

Supplementary Material 15

Supplementary Material 16

Supplementary Material 17

Supplementary Material 18

Supplementary Material 19

Supplementary Material 20

## Data Availability

All data generated or analysed during this study are included in this published article and its supplementary information files.

## Abbreviations

ZOL: Zoledronic acid
OP: Primary osteoporosis
APR: Acute phase adverse reactions
ROC: Receiver operating characteristic curve
LASSO: Least absolute shrinkage and selection operator
BMI: Body mass index
NSAIDs: Nonsteroidal anti-inflammatory drugs
ANOVA: Analysis of variance
CI: Confidence interval
SOCS3: Suppressor of cytokine signaling-3
AUC: Area under the curve
DCA: Decision curve analysis
OC: Osteocalcin
25 ( OH ) D: 25-hydroxyvitamin D
